# Efficacy of probiotics, paraprobiotics, and postbiotics in colorectal cancer cell line and their role in immune response

**DOI:** 10.1590/1806-9282.20240226

**Published:** 2024-07-19

**Authors:** Gülçin Alp Avci, Ülkü İrem Yilmaz, Emre Avci

**Affiliations:** 1 University of Health Sciences, Faculty of Gulhane Dentistry, Department of Basic Medical Sciences – Ankara, Turkey.; 2 University of Health Sciences, Gülhane Vocational School of Health, Department of Pathology – Ankara, Turkey.; 3 University of Health Sciences, Faculty of Gulhane Pharmacy, Department of Biochemistry – Ankara, Turkey.

**Keywords:** Probiotics, Cytotoxicity, Antimicrobial, Immunity

## Abstract

**OBJECTIVE:**

The aim of this study was to reveal certain features (anti-tumor/microbial activities) of postbiotics and heat-inactivated paraprobiotics obtained from two different bacteria with determined probiotic properties, which are thought to contribute to human health.

**METHODS:**

In the study, Lactobacillus reuteri ENA31 and L. rhamnosus GAA6 strains were used. Supernatants of postbiotically active cultures were used. Paraprobiotics were obtained by exposing probiotic bacteria to high temperatures. The cytotoxic effects of probiotics, paraprobiotics, and postbiotics were evaluated by the MTT method. IL-1/-10/-12/-13, TNF-α, IFN-γ, and neopterin parameters were determined via the ELISA method in immunity studies.

**RESULTS:**

It was detected that biotics had a cytotoxic effect on cancer cells with rising concentrations (paraprobiotic<probiotic<postbiotics, respectively). Intercalarily, with these biotic applications, a decline in the values of IL-1, IFN-γ, TNF-α, and neopterin and a rise in the values of IL-10/-12/-13 were observed in cancer cells.

**CONCLUSION:**

Our study shows that biotics, which are widely used and beneficial to health, are also available for use in immunocompromised individuals. The resulting paraprobiotics and postbiotics will both increase the conscious use of probiotics and provide the opportunity for use in immunocompromised individuals.

## INTRODUCTION

In the last few years, the use of structures known as paraprobiotics, which are non-living microorganisms, and postbiotics, which are metabolic by-products from bacteria to the external environment or resulting from the breakdown of bacteria, have become widespread as alternatives to probiotics. The crucial role of probiotics in maintaining intestinal homeostasis and in some gastrointestinal system disorders has been known for a long time, but there are still some pathways in the underlying mechanism that remain unclear^1^. Additionally, viability checks limit their use in the food and pharmaceutical industries. For this reason, the focus of studies is increasingly shifting from live probiotic microorganisms to non-living paraprobiotics, and biomolecules derived from probiotics, that is, postbiotics^1,2^. Postbiotics are a complex product of metabolic products with structural properties, such as enzymes, proteins, short-chain fatty acids, vitamins, peptides, and organic acids, secreted by probiotics in cell-free supernatants^3,4^. Paraprobiotics are inactivated microbial cells containing probiotics, which are intact or lysed structures or crude cell extracts containing cell components such as peptidoglycans, teichoic acids, and surface proteins^4^.

Like probiotics, paraprobiotics and postbiotics have been reported to exert a range of strain-specific health- promoting activities in individuals, including maintaining intestinal regularity at the physiological level, enhancing immunomodulatory activity, reducing inflammation, and inhibiting tumor development. However, although there are many studies on the efficacy of these para-post biotics, the pathways or mechanisms through which they exert these effects have not been fully explained. For these reasons, in our study, we aimed to obtain heat- inactivated paraprobiotics and postbiotics released from probiotics that can be used instead of probiotics and to contribute to the elucidation of the process by revealing some of their properties (cytotoxic, antimicrobial, and antioxidant activities) that may benefit human health.

## METHODS

### Selection, isolation, and identification of possible probiotic properties

In this study, a total of 15 lactobacilli were isolated from 5 breastfed infants' fecal samples. Isolate studies used the method of Hadadji et al.^5^. Bacterial DNA was extracted from gram-positive and catalase-negative strains using a genomic DNA extraction kit (27F: 5'-AGAGTTTGATCCTGGCTCAG-3' and 1492R: 5'-AAGGAGGGTGATCCAGCC-3'). *L. reuteri* ENA31 and *L. rhamnosus* GAA6 strains were selected according to their possible probiotic characteristics in studies.

In the selection of probiotics, the acid resistance, bile tolerance, and EPS production of the strains were determined using the method applied by Alp and Aslım^6^. The disk diffusion method was used to determine the antibiotic susceptibilities of the strains. The study included the six most commonly used antibiotics, including ampicillin, gentamicin, vancomycin, tetracycline, chloramphenicol, and ofloxacin. The inhibition zone diameter was measured with the help of calipers, and the data were evaluated together with the Antimicrobial Drug Susceptibility Testing Conduct Standard.

### Preparation of paraprobiotics

Bacteria were grown in MRS medium at 37°C for 18–24 h and then centrifuged at 5,000 g at 4°C for 10 min to obtain pellets. Afterward, it was washed three times with a saline solution and suspended in distilled water. Suspended bacteria were killed by subjecting them to heat at 80°C for 20 min, and a pellet was obtained by centrifuging at 5,000 g at 4°C for 10 min. The resulting pellets were suspended in distilled water and then lyophilized^7^.

### Preparation of postbiotics (cell-free)

The strains were grown in MRS broth at 37°C for 18–24 h and centrifuged at 2,000 g for 10 min. The supernatant was lyophilized^8^.

The chemical compositions of postbiotics were determined by the GC-MS and identified using calculated linear retention indices and mass spectra with those reported in the National Institute of Standards and Technology (NIST) database 2005^7,8^.

#### Determination of exopolysaccharide and bacteriocin as a postbiotic

Exopolysaccharide production of the strains was determined using the method applied by Alp and Aslım^7^. For bacteriocin, cultures were centrifuged to remove the cells, and the pH value of the resulting supernatants was adjusted to 7 with NaOH. Notably, 50% ammonium sulfate was added and mixed at 4°C for 24 h, and after the mixture was centrifuged under the same conditions, the pellets were collected and suspended in 25 mL of 0.05 M potassium phosphate buffer. The bacteriocin was partially purified by adding 15 mL of a methanol/chloroform (1:2 v/v) mixture and incubating at 4°C for 1 h.

### In vitro cell culture studies

The MTT method was utilized in cytotoxic studies. The viability of treated cultures with <70% test extract compared to untreated control cultures was considered to have cytotoxic effect according to ISO 10993-5.

### Determination of cellular immunity

In our study, IL-1/-10/-12/-13, TNF-α, IFN-IFN-γ, and neopterin parameters were examined using a commercially available enzyme-linked immunosorbent assay (ELISA, Rel Assay, Türkiye) kit.

### Statistical analysis

Differences were determined by applying one-way ANOVA and Tukey analysis using the data obtained from the studies using the IBM SPSS 22.0 statistical program, and the results were shown as mean±standard deviation. The statistical significance value was accepted as p<0.05. The parameters included in the research studies, total antioxidant capacity and scavenging activities of DPPH free radicals, were evaluated logarithmically on a graph.

## RESULTS

### Selection of bacteria

In our study, based on the probiotic properties of 15 lactobacilli isolated from the fecal samples of 5 breastfed babies, the 2 strains (L. rhamnosus GAA6 and L. reuteri ENA31) with the highest acid resistance (pH 3.0; 8.54±0.09 and 7.98±0.07, respectively), bile resistance (0.3%; 7.54±0.05 and 7.20±0.02, respectively), and EPS production (101.24 mg/L and 86.45 mg/L, respectively) were selected for use in other studies. Antibiotic sensitivity was also considered in strain selection: ampicillin (15.21±0.12), gentamicin (7.45±0.08), vancomycin (5.70±0.02), tetracycline (17.10±0.20), chloramphenicol (18.10±0.31), and ofloxacin (5.25±0.03).

### Exopolysaccharide and bacteriocin production

It was determined that the EPS production of the GAA6 strain (109.68 mg/L) was higher than the EPS production of the ENA31 strain (94.26 mg/L). The antimicrobial activity of the bacteriocin obtained from ENA31 and GAA6 was observed against some pathogenic bacteria. The findings obtained are presented in Table 1.

**Table 1:** Effect of bacteriocin obtained from Lactobacillus reuteri and Lactobacillus rhamnosus against gram-negative and gram-positive bacteria.

Zone of inhibition (mm)					
Bacteriocin	Gram-positive bacterial strains		Gram-negative bacterial strains		
	S. aureus ATCC 25923	E. faecalis ATCC 29212	P. aeruginosa ATCC 27853	E. coli ATCC 25922	B. subtilis ATCC 6633
L. reuteri	18.4±0.4	14.7±0.3	11.4±0.9	29.1±2.4	12.6±0.8
L. rhamnosus	19.6±1.6	14.9±0.8	11.8±1.1	29.4±2.1	13.8±0.2

### Cytotoxicity

**Figure 1:**
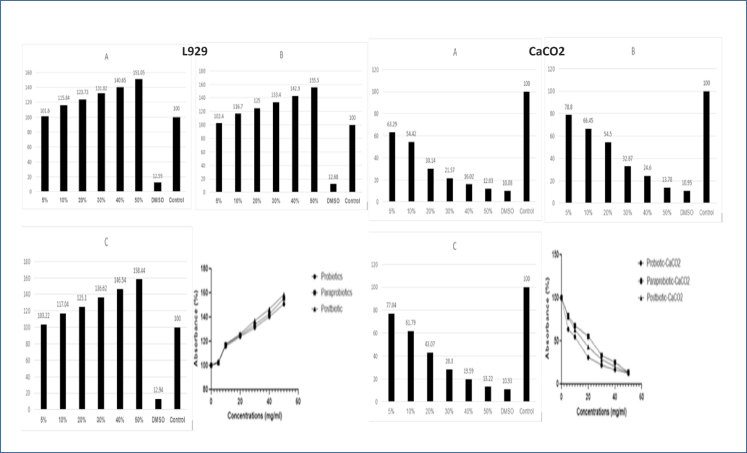
Effect of GAA6 on L929 and CaCO_2_ cell lines viability of probiotics (A), paraprobiotics (B), and postbiotics (C).

In our study, the proliferative effect of both GAA6 as a probiotic and postbiotics and paraprobiotics obtained from it was determined in the L929 cell line, which was included as the control group. It has been determined that GAA6 and the postbiotics and paraprobiotics derived from it have a cytotoxic effect on the cancer cell CaCO2 in parallel with the increasing concentration, and this effect is more visible in postbiotics and paraprobiotics (Figure 1).

**Figure 2:**
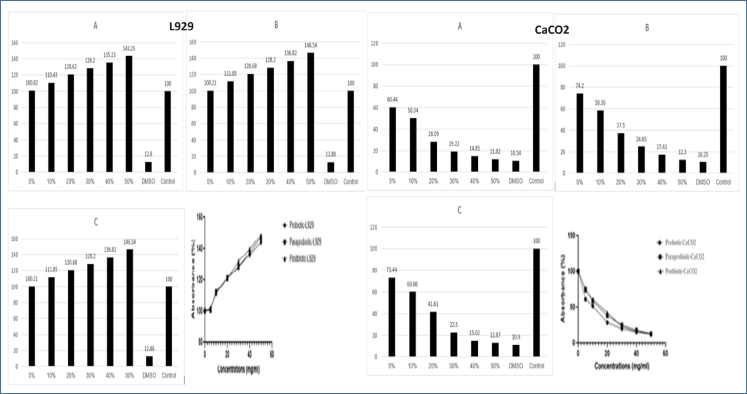
Effect of ENA31 on L929 and CaCO_2_ cell lines viability of probiotics (A), paraprobiotics (B), and postbiotics (C).

In our study, the proliferative effect of both ENA31 as a probiotic and postbiotics and paraprobiotics obtained from it was determined in the L929 cell line, which was included as the control group. It has been determined that ENA31 and the postbiotics and paraprobiotics derived from it have a cytotoxic effect on the cancer cell CaCO_2_ in parallel with the increasing concentration, and this effect is more visible in postbiotics and paraprobiotics (Figure 2).

### Determination of cellular immunity

A decrease in the levels of inflammatory cytokines (IL-1, TNF-α, and IFN-γ) and neopterin was determined in the control and the cells treated with probiotics, postbiotics, and paraprobiotics. On the other hand, an increase in the levels of IL-10/-12/-13 was detected. TNF-α level was 3.12±1.7 pg/mL in control-CaCO2 cells; after the addition of paraprobiotics, postbiotics, and probiotics, it was determined to be 2.54±0.4, 2.59±0.1 and 2.81±0.6 pg/mL, respectively. While the IFN-γ value was 1.24±0.6 pg/mL in control-CaCO_2_ cells, it was observed that it decreased to 1.06±0.2, 1.10±0.1 and 1.10±0.2 pg/mL with the addition of paraprobiotics, postbiotics, and probiotics, respectively. While the IL-1 value was 0.97±0.7 pg/mL in control-CaCO_2_ cells, it was determined to be 0.92±0.3, 0.86±0.1, and 0.79±0 pg/mL with the addition of paraprobiotics, postbiotics, and probiotics, respectively. While the IL-10 value was 2.21±1.0 pg/mL in control-CaCO_2_ cells, it was raised to 2.66±1.0, 2.51±0.8, and 2.34±1.2 pg/mL, respectively, with the addition of paraprobiotics, postbiotics, and probiotics. While the IL-12 value in control-CaCO_2_ cells was 2.05±0.9 pg/mL, with the addition of paraprobiotics, postbiotics, and probiotics, it was determined to be 2.48±1.7, 2.28±1.1, and 2.21±1.4 pg/mL, respectively. While the IL-13 value was 0.94±0.3 pg/mL in control-CaCO_2_ cells, it increased to 1.34±1.7, 1.25±1.4, and 1.14±1.6 pg/mL with the addition of paraprobiotics, postbiotics, and probiotics, respectively.

## DISCUSSION

In this context, studies reveal that postbiotics and paraprobiotics exhibit bioactivities such as anti-inflammatory, immunomodulatory, antioxidant, and antimicrobial as well as anticarcinogenic benefits^9-11^. Lactic acid bacteria have been reported to be beneficial to health by secreting lactic acid, peptidoglycan, bacteriocin, and metabolites that may be postbiotics. For example, it is reported that lactate and short-chain fatty acids produced by fermentation are postbiotics and affect the anti-inflammatory and anticarcinogenic properties of the intestine^12^. Recently, especially EPS, bacteriocins, and biosurfactants have attracted attention as postbiotics. Studies emphasize the importance of biotics with high EPS production capacity, biosurfactant production, and bacteriocin activity. Biotics with these features will contribute much more to the benefit of human health.

It has been reported in the literature that paraprobiotics inactivated by heat treatment do not interfere with the increase in the production of immune-supporting cytokines in macrophages, and thus the paraprobiotic has a positive effect on immunity^13,14^. One of the health problems for which the effects of paraprobiotics and postbiotics have been investigated is colon cancer. Several paraprobiotic fractions (heat-inactivated cells, cell walls, peptidoglycan, and cytoplasmic structures) derived from *Lactobacillus* spp. that have antiproliferative effects against human cancer cells are reported in the literature^13^. In a study conducted by Cicenia et al. on the effect of postbiotics on the colon, it was stated that smooth muscle cells in the human colon, *L. rhamnosus*, protected postbiotic chemicals that mediate postbiotic reactions from lipopolysaccharides that cause myogenic damage. Studies have concluded that paraprobiotics and/or their cell wall extracts can alleviate inflammation in ways similar to those of live bacteria^14^. In a study by Chuah et al., different postbiotics derived from *L. plantarum* strains exhibited selective cytotoxic activity through antiproliferative effects and induction of apoptosis against cancer cells, showing that they are strain-specific and cancer cell type-specific^9^.

The immune system neutralizes cellular and humoral agents through different mechanisms. The literature reveals that the gut microbiome has a vital role, especially in the development of the host's immune system and the regulation of metabolic events^15^. Studies prove that paraprobiotics and postbiotics are effective on the immune system. *Bifidobacterium* spp. act against active ulcerative colitis and exacerbations of this disease. It has been observed that the application of paraprobiotic *Bifidobacteria* in fermented milk triggers the production of IL-10, an anti-inflammatory cytokine, and suppresses the secretion of IL-8, a pro-inflammatory cytokine, in epithelial cells^15^. Riaz et al. studied the cell-free supernatant of the liquid culture of three *L. rhamnosus* strains isolated from human breast milk and showed the antioxidant activities against radicals^16^. Song et al. studied the use of both live and heat-inactivated samples conducted with *L. brevis* B13-2 and showed that both forms showed antioxidant activity, while the paraprobiotic form exhibited both stability and immunomodulatory activity. It has been shown that they can be used as functional components^17^. Balzaretti et al. reported that an exopolysaccharide derived from *L. paracasei* DG as a postbiotic inhibited proinflammatory cytokines in the human monocytic cell line^18^. Qi et al. found that different postbiotic compounds, derived from *L. rhamnosus* GG, including surface layer protein, genomic DNA, and unmethylated cytosine- phosphate-guanine containing oligodeoxynucleotides, were activated by mitogen-activated protein kinases in lipopolysaccharide- stimulated mouse macrophage cells^19^.

## CONCLUSION

Hundreds of probiotic products are sold and used commercially around the world. However, although these products are not products of our country, they are brought from abroad and are widely used because they are beneficial to health, without their scientific data being researched in detail with academic studies. With the data obtained within the scope of our study, both the acquisition of new probiotics and the diversification of existing products—extending the shelf life—can be achieved. Considering all these study results, postbiotics and paraprobiotics show beneficial activity in the health process in the regulation of the immune system in the host. It is an important finding that this situation will be a safer alternative for premature infants, elderly individuals, and transplant patients, especially for immunocompromised and affected individuals, and some of the disadvantages that probiotics may cause in these individuals will be eliminated.
